# Rapid Protein–Ligand Affinity Determination by Photoinduced Hyperpolarized NMR

**DOI:** 10.1021/jacs.4c04000

**Published:** 2024-06-19

**Authors:** Matthias Bütikofer, Gabriela R. Stadler, Harindranath Kadavath, Riccardo Cadalbert, Felix Torres, Roland Riek

**Affiliations:** † Institute for Molecular Physical Science, Vladimir Prelog Weg 2, 8093 Zürich, Switzerland; ‡ NexMR AG, Wiesenstrasse 10A, 8952 Schlieren, Switzerland

## Abstract

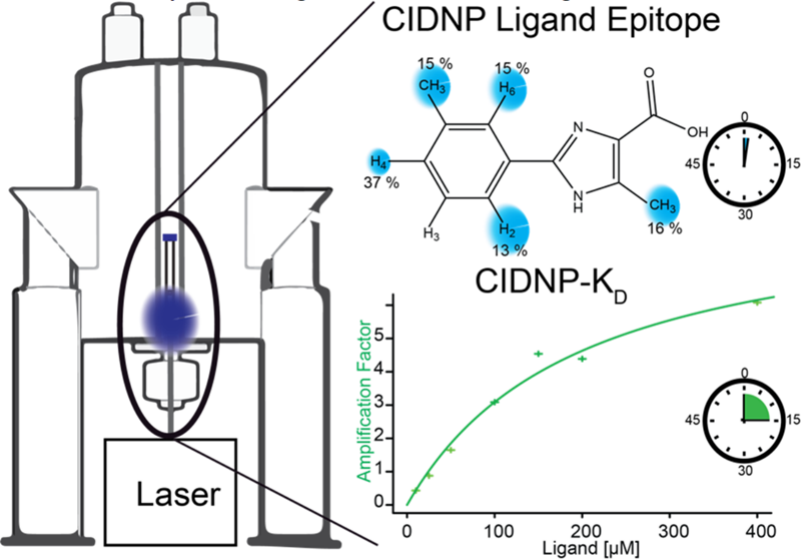

The binding affinity determination of protein–ligand complexes is a cornerstone of drug design. State-of-the-art techniques are limited by lengthy and expensive processes. Building upon our recently introduced novel screening method utilizing photochemically induced dynamic nuclear polarization (photo-CIDNP) NMR, we provide the methodological framework to determine binding affinities within 5–15 min using 0.1 mg of protein. The accuracy of our method is demonstrated for the affinity constants of peptides binding to a PDZ domain and fragment ligands binding to the protein PIN1. The method can also be extended to measure the affinity of nonphoto-CIDNP-polarizable ligands in competition binding experiments. Finally, we demonstrate a strong correlation between the ligand-reduced signals in photo-CIDNP-based NMR fragment screening and the well-established saturation transfer difference (STD) NMR. Thus, our methodology measures protein–ligand affinities in the micro- to millimolar range in only a few minutes and informs on the binding epitope in a single-scan experiment, opening new avenues for early stage drug discovery approaches.

The design of novel drug candidates relies heavily on protein–ligand binding affinity determinations. The accurate assessment of such affinities is critical for medicinal chemists to select the most potent small molecules within hit or lead series and advance the design of a drug candidate. However, the traditional methods used to determine protein–ligand affinities, such as isothermal titration calorimetry (ITC),^1^ surface plasmon resonance (SPR),^2^ or thermal shift assay (TSA),^3^ often have time-consuming protocols, resource-intensive requirements, and complicated assay development or are not applicable for weak binders. Microscale thermophoresis (MST) and differential scanning fluorimetry (DSF)^4^ are relatively new methods using little sample and requiring no target immobilization, with the limitation that the sensitivity for weak-binding fragments is low.^5,6^ Also, since both methods rely on fluorescence, adding a fluorescence label to the protein target is often necessary, and parameters like molecule size, charge, unfolding temperature, or hydration shell can affect the data interpretation.

Nuclear magnetic resonance (NMR) provides a wide range of methods that can be used to derive protein–ligand complex structures and map binding epitopes and measure protein dynamics and binding kinetics and is particularly well-suited to weak-affinity interactions.^7−9^ However, a method such as heteronuclear single quantum coherence (HSQC),^10^ which can provide information on both the binding site and the dissociation constant (*K*_D_), requires the isotope labeling of 2–12 mg of protein (assuming a 20 kDa protein) and long experimental time. Ligand-observed methods such as saturation transfer difference (STD)^11^ can also inform the *K*_D_(7) and the ligand epitope^12^ without the need for isotope labeling; however, the total measurement time for one *K*_D_ is several hours. Recently, Monaco et al. presented an elegant manner of obtaining an STD-*K*_D_ within a single sample, whereby the ligand is deposited as a drop and diffuses to create a gradient.^13^ This approach is economical in the sample material, which is particularly attractive for later lead series. Its usage at earlier stages, where one needs to characterize dozens of molecules, is challenging due to the time-consuming manual sample preparation. Moreover, it still suffers from the relatively low sensitivity of STD-NMR, requiring experimental times on the order of hours for a single-affinity determination. Alternatively, recent work leverages transverse relaxation rates to derive the affinity for protein–ligand interactions.^14^ The method provides a quick way to first rank hits obtained by screening and later measure the affinity. However, transverse relaxation rate measurements require the acquisition of decay plots for each titration point, again leading to experimental times in the order of hours.

To accelerate the acquisition time, hyperpolarization methods improving NMR sensitivity, such as dynamic nuclear polarization (DNP),^15^ para-hydrogen-induced polarization (PHIP),^16^ and signal amplification by reversible exchange (SABRE),^17^ have been successfully delivering impressing signal-to-noise enhancement (SNE), limiting the need for always more powerful magnetic fields.^18^ However, these methods add instrumental complexity, limiting scalable adoption. Furthermore, the hardware complexity challenges the experimental repeatability required for the quantitative analysis in *K*_D_ determination.^19−22^

Recently, we demonstrated how photochemically induced dynamic nuclear polarization (photo-CIDNP) can boost NMR sensitivity by simply illuminating the sample from the bottom of a cryoprobe, which is fully compatible with commercial autosamplers, making photo-CIDNP NMR experiments recordable in an automatic manner.^23^ Such an automated platform is attractive due to its simple setup, i.e., a laser coupled to the NMR through an optic fiber, and photo-CIDNP is performed at mild sample conditions, i.e., in aqueous buffer and at room temperature.^24,25^ Photo-CIDNP facilitates the signal-to-noise enhancement of ligands by 20–100-fold, increasing the experimental throughput with experimental time within seconds. Another advantage is the reduction of sample concentration with ligand concentrations down to 5–10 μM and protein concentration down to 1–2 μM.^24^ This work demonstrates how hyperpolarization, specifically photo-CIDNP, can analyze protein–ligand interactions quantitatively and derive affinities within 10–15 min. Like STD-NMR *K*_D_ and T_2_-*K*_D_, our method eliminates the need for isotope labeling or other assay development, yielding a streamlined, cost-effective workflow highly compatible with automated high-throughput screening campaigns. However, as the method relies on the ligand being in the fast-exchange regime, only affinities higher than 10–20 μM can be determined with CIDNP-*K*_D_. Another benefit of the similarity between our method and STD-NMR is the possibility of identifying the ligand’s binding epitopes.^12^ Indeed, the selective relaxation of the hyperpolarized ligand protons depends on the surrounding spin density, like STD-NMR, giving insights into the binding mode of the ligand. We demonstrate the correlation between these two methods and the possibility of augmenting the data gathered from photo-CIDNP screening and obtaining early structural information.

## Theory

Photo-CIDNP hyperpolarization is achieved when a small molecule selectively reacts with a photosensitizer after shining light into the sample. In a magnetic field, the excited triplet-state photosensitizer and the molecule of interest form a radical pair for which the singlet–triplet mixing frequency after recombination depends on the nuclear spin state. The photochemical reaction yields back the two molecules in their original form but with an out-of-Boltzmann equilibrium distribution of the nuclear spin state, resulting in selective hyperpolarization.^26−28^

**Scheme 1:**
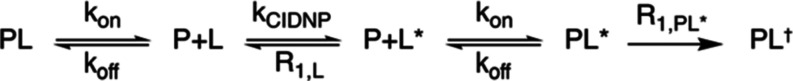
Reaction Mechanism Underlying the CIDNP-*K*_D_ Method During light irradiation of the sample, the ligand L undergoes hyperpolarization with the photo-CIDNP rate constant *k*_CIDNP_ to form the hyperpolarized ligand L*, which relaxes with the longitudinal relaxation rate R_1,L_. In the presence of a protein P, either L or L* can bind to P with the rate constant *k*_on_ to form the complex PL or PL*, respectively. The complexes can dissociate with the off-rate *k*_off_ or in the case of the hyperpolarized complex PL* relax with the selective longitudinal relaxation rate R_1,PL*_ to PL†, which is chemically identical to PL but whose identity is given a separate notation because it is this species whose concentration determines the observed signal reduction.

The theoretical basis of the photo-CIDNP dissociation constant determination method (denoted therein CIDNP-*K*_D_) lies in the selective polarization of the ligand and subsequent protein binding, which is depicted in Scheme 1. Upon excitation, the hyperpolarized ligand L* can relax to its thermal equilibrium state L or bind to the protein P to form the complex PL*. The complex can either dissociate or relax to PL†, where the ligand is at thermal equilibrium polarization, with a selective longitudinal relaxation rate (R_1,PL*_).^24^

**Figure 1:**
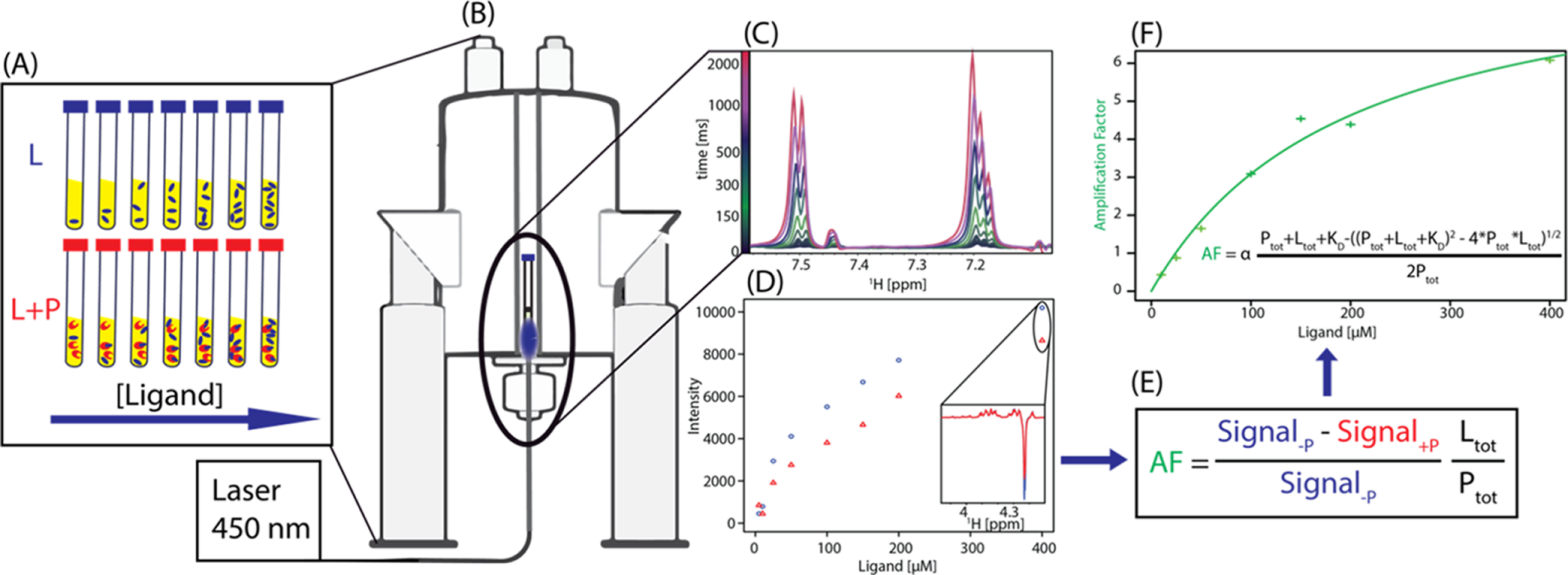
CIDNP-*K*_D_ titration workflow. (A) Sample preparation of the photosensitizer (indicated in yellow) and the photo-CIDNP-active compound in the absence and presence of the target protein. (B) Modifying the cryoprobe with Cryolight allows the measurement of the samples from the top and enables the use of an autosampler. (C) Light-induced hyperpolarization builds up after 2 s, and (D) signal reduction of the hyperpolarized ligand at different concentrations is observed in the presence of the target protein. Signal intensities are represented for irradiation times after reaching the steady state (>100 ms). (E) Eq 4 converts the signal intensity into the concentration-dependent amplification factor (F), fitted with eq 5 to obtain the dissociation constant.

The fast selective longitudinal relaxation rate, R_1,PL*_, is the primary determinant for the observed signal reduction effect upon which CIDNP-*K*_D_ is built.^24^ Other sources of signal reduction, like the reaction of the radicals with the protein or collision of triplet-state dye with the protein, are not ligand concentration-dependent and thus will not influence the *K*_D_ determination. They were also not observed in the system under study here as evaluated by nonbinding ligand photo-CIDNP experiments.^24,25^ Furthermore, two *K*_D_ determinations with two different irradiation times would evaluate the potential of photoactivated covalent ligand–protein binding, which was not observed in the case study presented. Thus, the photo-CIDNP signal intensity in the presence of protein is proportional to the concentration of the free hyperpolarized ligand
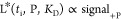
1where L*(*t*_i_, P, *K*_D_) is the irradiation time-, protein concentration-, and affinity-dependent hyperpolarized ligand concentration, respectively, and signal_+P_ is its corresponding signal intensity (Figure 1D, red) in the 1D NMR spectrum.

The signal intensity of the ligand in the absence of the protein is proportional to the total ligand concentration L*_tot_
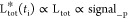
2where L*_tot_(*t*_i_) is the irradiation time-dependent hyperpolarized ligand concentration in the absence of protein and signal_–P_ is its corresponding NMR spectrum intensity (Figure 1C,D, blue). Therefore, the signal loss upon protein addition is proportional to PL† (*t*_i_, L, P, *K*_D_), forming upon binding of L* to P and R_1,PL*_ relaxation. PL† can be expressed as the fraction-bound ligand population
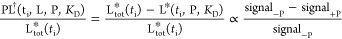
3By multiplying eq 3 with the excess of ligand L_tot_/P_tot_ and the proportionality factor α, the fraction-bound ligand is converted into the fraction-bound protein population
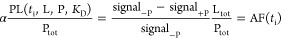
4with AF(*t*_i_) being the time-dependent amplification factor, similar to STD-NMR.^11^

For both STD-NMR and CIDNP-*K*_D_, there is an initial buildup of the signal at the start of the irradiation. However, the mechanisms underlying it and, thus, the kinetics of the signal buildup are different between the techniques. In STD-NMR, the saturation of L starts to build up as soon as the irradiation of the protein begins because preformed PL complexes can be immediately saturated. However, in CIDNP-*K*_D_, the preformed PL complexes are not CIDNP-active. Therefore, a free L must first be hyperpolarized and then bind to P before any signal reduction is observable. However, after only a few hundred milliseconds of irradiation, all kinetic rates reach a steady state, as indicated in Figure S1B. Therefore, we will use the AF(*t*_i_) corresponding to the steady state in contrast to STD-NMR, where one needs to measure the initial growth of the amplification factor to account for the ligand rebinding effects, which are significant at low ligand-to-protein ratios;^7^ there is no need for an initial amplification factor in CIDNP-*K*_D_. During the photo-CIDNP process, only 0.1–1% of the ligand becomes hyperpolarized such that even at low ligand-to-protein ratios, the rebinding of L* is not a significant factor. Therefore, one needs to only assess the time to reach a steady-state amplification factor AF_ss_, whose value is measured at a series of ligand concentrations, and then fit eq 5 to determine the proportionality factor α and the dissociation constant *K*_D_ (Figure 1D,E)
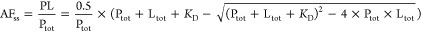
5Affinities are obtained with the CIDNP-*K*_D_ method as follows. The ligand of interest and the photosensitizer are prepared as titration series in the presence and absence of the target protein (Figure 1A) and measured with the Cryolight setup (Figure 1B,^23^), which allows the automatization with an autosampler. The photo-CIDNP signal builds up within 2 s (Figure 1C). It allows us to measure the irradiation time-dependent photo-CIDNP buildup of the ligand NMR signal in the presence and absence of protein in under a minute per sample (Figure S1A). These signal intensities are converted into AF(*t_i_*) with eq 4 (Figure S1B), giving an individual titration data point in the corresponding ligand titration curve (Figure S1C). The experiment is repeated at different ligand concentrations to build the titration curve fitted using eq 5, yielding the *K*_D_ (Figure 1E,F). The entire measurement of all the photo-CIDNP spectra of 7 titration points (14 measurements) takes about 7 min in measurement time, plus the time to exchange the samples with the autosampler (Figure 1D).

**Scheme 2:**
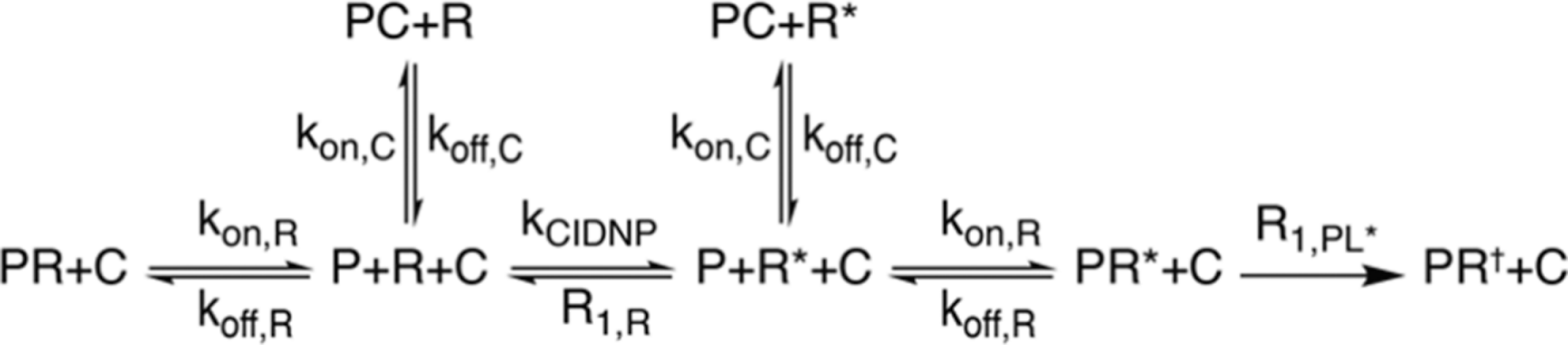
Interaction Scheme of the CIDNP-*K*_D_ Competition Assay The competitor C can form with the free protein P the complex PC in the presence of the reporter R and R*, reducing the chance that PR and PR* form. Therefore, the signal reduction of PR† through selective R_1,PL*_ relaxation is reduced, and the NMR signal of R in the presence of C is larger.

Photo-CIDNP hyperpolarization is limited to a particular chemical space of ligands that contain heteroaromatics and heterosubstituted aromatic rings.^24,29^ To address the challenge of limited chemical space, we extend CIDNP-*K*_D_ to measure the affinity of nonpolarizable ligands through competition with a polarizable reporter ligand R, whose affinity *K*_D,R_ is known as depicted in Scheme 2. By adding a competitor C, the concentration and photo-CIDNP signal of the reporter ligand R* will increase, indicating the reduced amount of the complex PR* and, by extension, PR.^30^

To calculate the concentration of the ligand–protein complex in the presence of the binding competitor, the ratio of the amplification factors in the presence and absence of the competitor
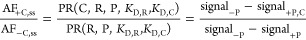
6is used to obtain PR(C, R, P, *K*_D,R_, *K*_D,C_).^31^ By fitting this value against the competitor concentration C with the cubic equation^32^
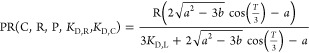
7where
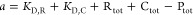
8
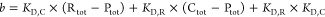
9
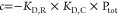
10and
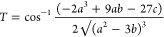
11one obtains the affinity constant *K*_D,C_ of competitor C.

## Results

### *K*_D_ Determination of Peptides That Bind to a PDZ2 Domain

The human tyrosine phosphatase 1E protein (hPTP1E) interacts with the Ras-associated guanine nucleotide exchange factor 2 (RA-GEF2) via its second PDZ domain (PDZ2) to modulate various cellular processes, including cell proliferation.^33^ We studied the interaction of the PDZ2 domain of hPTP1E with the peptide segment EQVSAV, the consensus interacting motif of RA-GEF2.^34^ We derived the peptides WSAV, WVSAV, WQVSAV, and WEQVSAV to evaluate the influence of each residue on the PDZ2-EQVSAV complex affinity and establish our CIDNP-*K*_D_ method. The range of peptide lengths was chosen to yield a range of affinities, with longer sequences expected to show stronger affinities. The N-terminal tryptophan was added as a well-known photo-CIDNP-active tag.^35^ It is also possible to label the peptides with different amino acids that are well known for their capacity to yield SNE through photo-CIDNP, such as tyrosine, histidine, methionine, and N-methyl lysine.^36^

**Figure 2:**
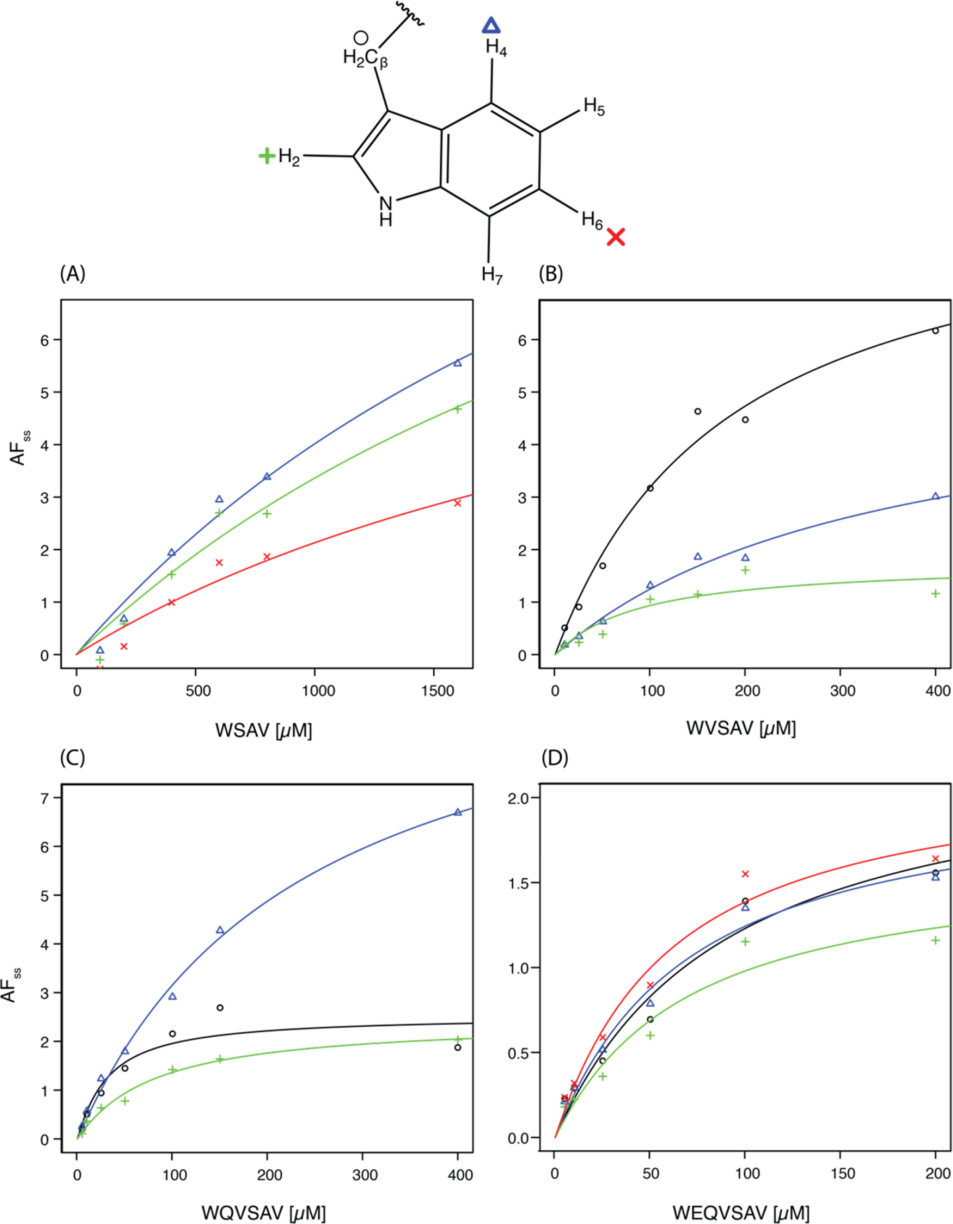
Affinity determination of different peptides binding to the hPDZ2 domain by CIDNP-*K*_D_ within a few minutes. Ligand titration of (A) WSAV, (B) WVSAV, (C) WQVSAV, and (D) WEQVSAV against the human tyrosine phosphatase PDZ2 domain (20 μM for WSAV, 10 μM for others) as described in Figures 1 and S1. The plots contain the titration curves for all protons of the tryptophan residue that undergo signal enhancement through photo-CIDNP and display sufficient quenching upon protein binding. The individual data points are measured with single-scan experiments with laser irradiation durations where *t* > *t*_ss_: (A) 1500 ms, (B) 2000 ms, (C) 2000 ms, and (D) 2000 ms.

Figure 2 presents the CIDNP-*K*_D_ titration curves for each peptide with 20 μM PDZ2 in (A) and 10 μM PDZ2 in (B to D). The curves were obtained using the workflow described in the introduction for each proton of tryptophan that has sufficient photo-CIDNP signal enhancement and signal reduction upon binding (Figure S2).

We compared the affinities of the peptides obtained via CIDNP-*K*_D_ with the state-of-the-art 2D [^1^H, ^15^N]-HSQC titration experiments measured with 30 μM PDZ2 (Figure S3). Table 1 shows a summary of all measured affinities with the two methods.

**Table 1:** Dissociation Constants (*K*_D_) Measured by Titration Series of CIDNP-*K*_D_ Measurements, Competition CIDNP-*K*_D_ Measurements, and [^1^H, ^15^N]-HSQC of Indicated Peptides Binding to the PDZ2 Domain

	CIDNP-*K*_D_ [μM]				
	H_β_	H_2_	H_4_	H_6_	[^1^H,^15^N]-HSQC–CSP [μM] NH of A74 (range)^a^
WSAV	-b	3300 ± 2300	3100 ± 1500	3100 ± 3200	1320 ± 160 (200–4000)
WVSAV	170 ± 40	80 ± 60^c^	330 ± 80	-c	108 ± 10 (50–400)
WQVSAV	27 ± 16	74 ± 14^c^	230 ± 30	-c	68 ± 11 (20–280)
WEQVSAV	82 ± 33	63 ± 28	63 ± 16	55 ± 17	16 ± 5 (10–70)
QVSAV	580 ± 30	-d	-b	-d	155 ± 10 (90–750)
ENEQVSAV	35 ± 2	-d	91 ± 6	-d	17 ± 7 (10–80)

a *K*_D_ range found of all residues showing chemical shift perturbation during [^1^H, ^15^N]-HSQC titration.

b No fit could be derived.

c Signal overlap of H_2_ and H_6_ disabled the latter’s use in CIDNP-*K*_D_ affinity determination.

d Not sufficient signal reduction of the reporter ligand upon protein addition. No competition CIDNP-*K*_D_ was obtained.

With the CIDNP-*K*_D_ method and observing the aromatic tryptophan protons H_2_, H_4_, and H_6_, the affinity of WSAV was elucidated to be 3100–3300 μM. No fit was derived for the H_β_ (Figure 2A). The 2D [^1^H, ^15^N]-HSQC ligand titration experiments induced chemical shift perturbations at several residues, yielding calculated affinities ranging from 200–4000 μM, a range that has been the topic of much controversy.^37^Table 1 lists only the data for the ^15^N–^1^H moiety of A74 because it is directly involved in the binding site at the C-terminal region of the peptides and should interact similarly with all four peptides.^38^ The analysis of the ^15^N–^1^H moiety of A74 yielded a *K*_D_ of 1320 ± 160 μM (Figure S3A), which is in the same order of magnitude as the CIDNP-*K*_D_ measurements (Table 1). Similarly, the [^1^H, ^15^N]-HSQC-derived *K*_D_ for the peptide WVSAV ranged from 50–400 μM with a *K*_D_ from the ^15^N–^1^H moiety of A74 of 108 ± 10 μM (Figure S3B). The affinities measured with CIDNP-*K*_D_ range between 80 and 330 μM for H_β_, H_2_, and H_4_ (Figure 2B). As the singlet of H_2_ and the triplet of H_6_ overlap (Figure S2B), the *K*_D_ from H_2_ must be interpreted cautiously, and it was impossible to analyze the *K*_D_ from H_6_.

The CIDNP-*K*_D_ for the peptide WQVSAV yielded affinities between 19 and 200 μM (Figure 2C) matching into the range of 27–230 μM measured by 2D [^1^H, ^15^N]-HSQC NMR and 68 ± 11 μM from A74 (Figure S3C). Lastly, the peptide WEQVSAV showed a CIDNP-*K*_D_ affinity of 55–82 μM (Figure 2D) compared to the affinity of 10–70 μM measured by 2D [^1^H, ^15^N]-HSQC NMR with 16 ± 5 μM *K*_D_ from A74 (Figure S3D).

**Figure 3:**
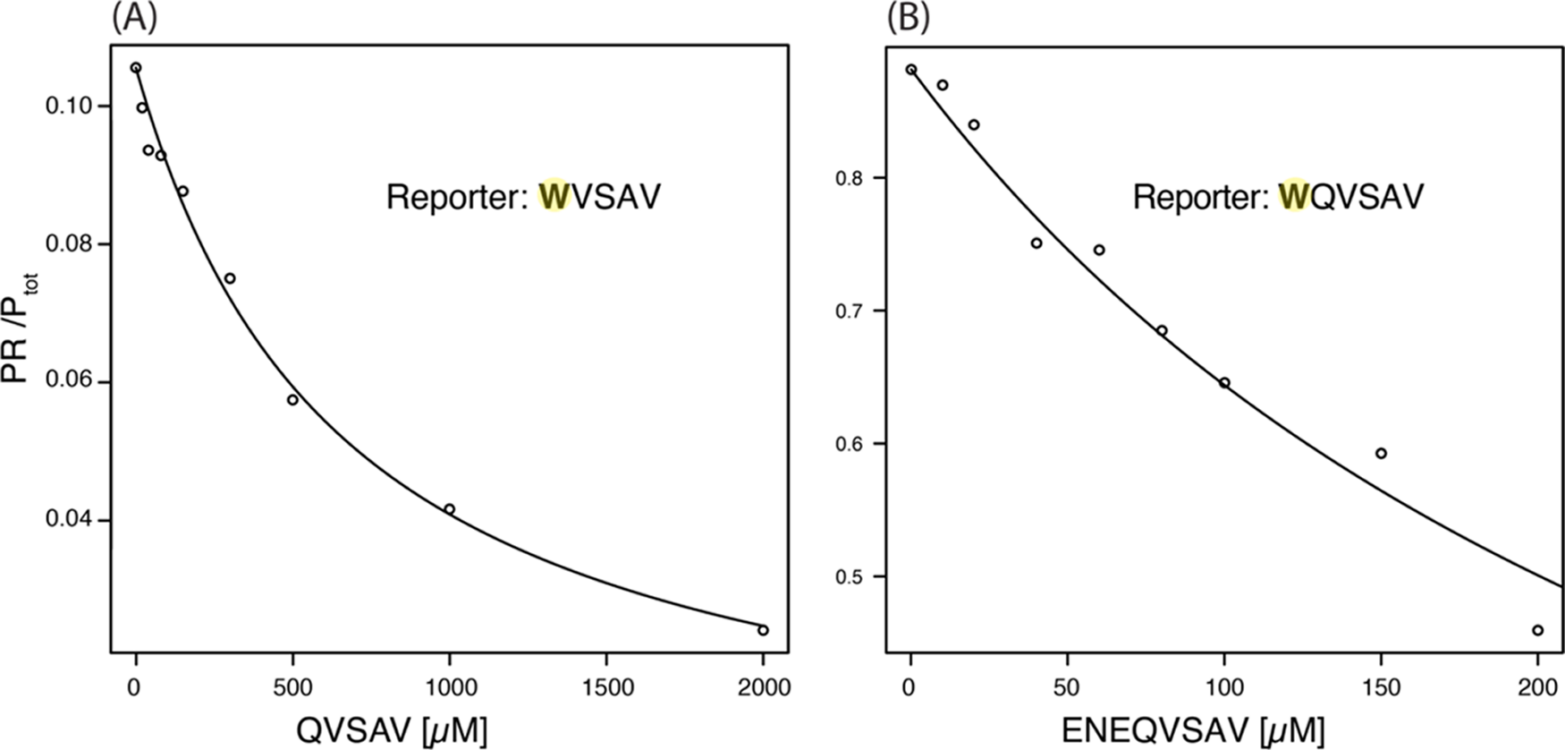
*K*_D_ determination of nonphoto-CIDNP-active peptides binding to a PDZ2 domain. Titration curves for (A) WVSAV (20 μM) bound to hPDZ2 (5 μM) with QVSAV and (B) WQVSAV (150 μM) bound to PDZ2 (10 μM) with ENEQVSAV. The bound reporter population is plotted, calculated from the hyperpolarized tryptophan H_β_ using eq 7. The points were used to fit with eq 8 using the known CIDNP-*K*_D_ values of 170 μM and 27 μM for WVSAV and WQVSAV, respectively. Each data point is measured with a single-scan experiment with a laser irradiation duration of (A) 1000 ms or (B) 2000 ms.

Next, we measured the affinity of the nonphoto-CIDNP-polarizable peptides, QVSAV and ENEQVSAV, using a photo-CIDNP competition assay, providing *K*_D,C_ values within 10–15 min measurement time. These competitor ligands lack a tryptophan residue, whereas the reporter ligands WVSAV and WQVSAV are photo-CIDNP-active due to the tryptophan tag. The hyperpolarized signal of WVSAV or WQVSAV in the presence of the PDZ2 domain was measured for increasing concentrations of the peptides QVSAV or ENEQVSAV, respectively. The reporter signal was converted with eq 7 to the bound reporter population PR/P_tot_. Figures 3A and S4A show the titration of QVSAV to the PDZ2 domain using the signals of H_β_ and H_4_, respectively, of the reporter ligand WVSAV. By fitting eq 8 and using the CIDNP-*K*_D_ of 170 μM and 330 μM for H_β_ and H_4_, respectively, the obtained *K*_D,C_ for the H_β_ signal of QVSAV was 580 ± 30 μM. No fit could be obtained for the H_4_ signal, but the data points indicate a similar trend, as shown in Figure S4A. The reference 2D [^15^N,^1^H]-HSQC chemical shift titration experiments of the peptide QVSAV yielded a *K*_D_ ranging from 90–750 μM, with 155 ± 10 μM from the ^15^N–^1^H moiety of A74, yielding a similar value to the competition CIDNP-*K*_D_.

Figures 3B and S4B display the CIDNP-*K*_D_ competition assay of ENEQVSAV titrated to WQVSAV and PDZ2, using the tryptophan H_β_ and H_4_ signals, respectively. Using an affinity constant of 27 μM (H_β_) and 230 μM (H_4_) for the reporter peptide WQVSAV, *K*_D,C_ values of 35 ± 2 μM and 91 ± 6 μM were obtained for ENEQVSAV, respectively. This finding agrees with the affinity determined by [^15^N,^1^H]-HSQC chemical shift perturbation experiments that yield a *K*_D_ range of 10–80 μM with 17 ± 7 μM for the ^15^N–^1^H moiety of A74 (Figure S3F).

### CIDNP-*K*_D_-Based *K*_D_ Determination of Fragments

**Figure 4:**
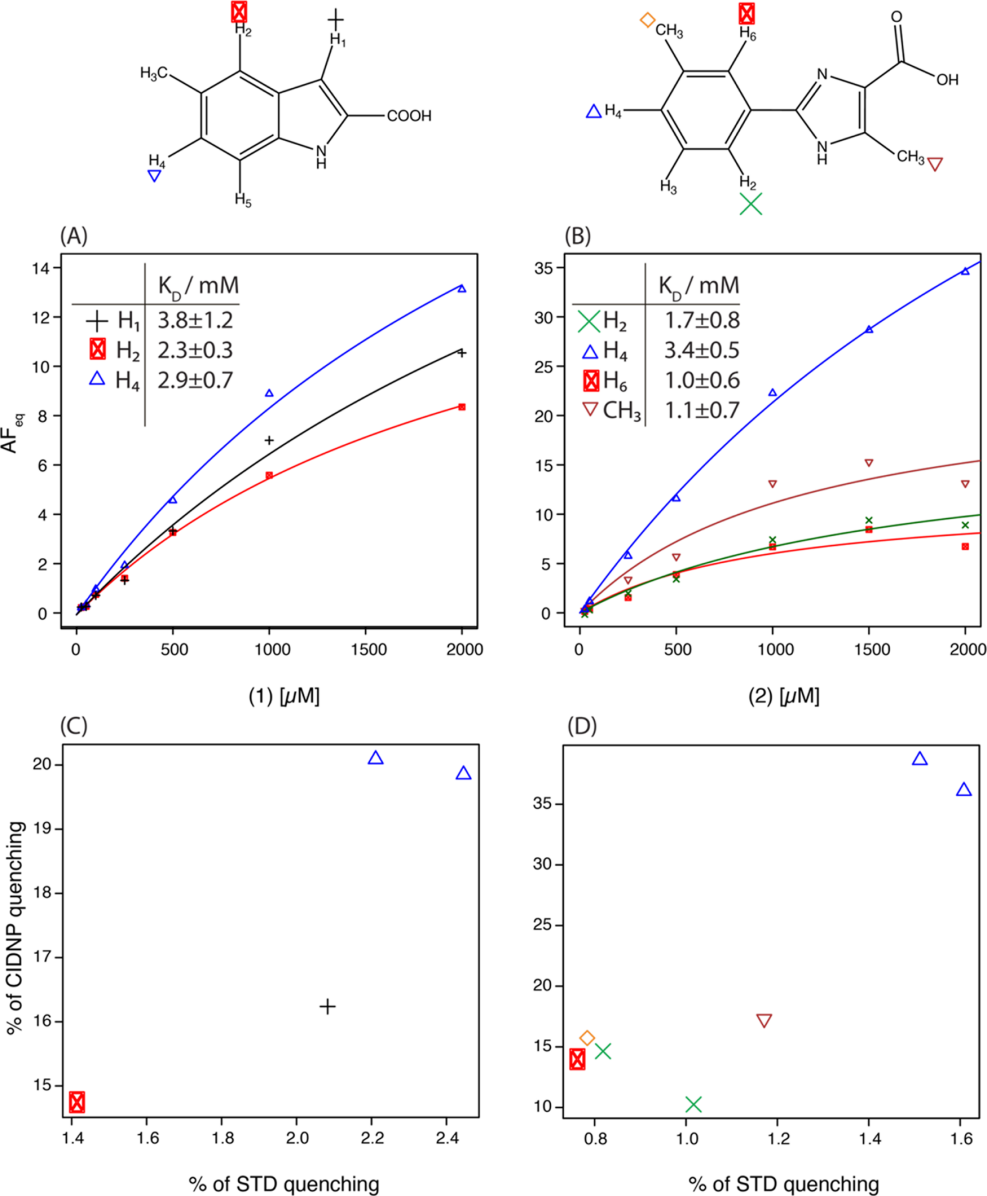
CIDNP-*K*_D_ determination and ligand-observed epitope mapping of fragments binding to PIN1. The CIDNP-*K*_D_ titration curves of 1 (A) and 2 (B) were measured with 20 μM PIN1. Each data point represents a single-scan experiment with a laser irradiation time of 2000 or 2000 ms for 1 and 2, respectively. (C, D): Normalized intensity of the photo-CIDNP difference spectrum is plotted against the normalized intensity of the STD difference spectrum for compounds 1 and 2. The measurements were conducted at 500 μM fragment and at 20 μM PIN1. The doublet peak of H_4_ from both fragments and H_2_ of 2 is presented as two peaks.

While CIDNP-*K*_D_ is conveniently applied to peptides that can be modified to be polarizable or that compete with a polarizable ligand, previous work from us demonstrated the capacity to design photo-CIDNP-compatible fragment libraries and perform screening for hit discovery, which we showcased with a screening against human PIN1.^24,25^ Besides its biological functions as the regulation of mitosis^39^ or protection against Alzheimer’s disease,^40^ the *cis*–*trans* isomerase is also overexpressed in human cancer cells, making it an attractive drug target.^41^ In the previously reported photo-CIDNP NMR screening of our photoinducible fragment library against PIN1, 20 hits out of 212 fragments were identified and validated. Most of these hits were very weak binders (>5 mM); therefore, we selected the two well-characterized hits (compounds 1 and 2), also identified during the photo-CIDNP NMR screening campaign, to show the applicability of the CIDNP-*K*_D_ method for small molecule fragments.^42,43^ Using the CIDNP-*K*_D_ methodology, the *K*_D_ was obtained for 1 and 2 for which we previously reported the affinity for PIN1.^24^Figure 4A,B shows the CIDNP-*K*_D_ titration curves of compounds 1 and 2, respectively. A titration curve was derived for each hyperpolarized proton after irradiation at 450 nm in the presence of 10 μM fluorescein, and the data were collected manually within 15 min for each fragment. While the different protons have distinct hyperpolarization yields, all provide consistent affinity constants. Each data point represents an average of several single-scan experiments with different laser irradiation durations. The CIDNP-*K*_D_ method yields binding affinities for both compounds in the 1–3.8 mM range. These results agree with our previously reported binding constants for compounds 1 and 2 to be 1.7 ± 0.2 and 1.5 ± 0.2 mM, respectively, using 2D [^15^N,^1^H]-HSQC-based titration curves of the PIN1 residue T174.^24^ Since the recent development of photoinducible fragment libraries^24^ comprising several hundreds of chemically diverse fragments and automated light-couple NMR platforms, it is possible to perform an NMR fragment screening in a few hours to a few days. It is, therefore, critical to quickly determine the affinity and assess which hits, among dozens, should be prioritized.

### CIDNP-Based Epitope Mapping

The quantitative ^1^H signal reduction upon binding to PIN1 (AF_ss_) is distinct for each ^1^H of compounds 1 and 2 (Figure S5C,D) because photo-CIDNP signal reduction depends strongly on the selective relaxation rate R_1,PL_, which in turn depends on the degree of cross-polarization with surrounding protons in the binding pocket. Therefore, this relaxation, similar to the nuclear Overhauser effect (NOE), has a distance dependency of r^–6^, and signals should decrease to different degrees according to their binding site environment and orientation.^11,44^ This permits the signal reduction of CIDNP-*K*_D_ NMR to be similar to STD epitope mapping^12^ or ligand orientation in the binding pocket.^45^ In support of this notion, Figure 4C,D shows the correlation between STD-NMR and CIDNP-*K*_D_ signal reduction of the individual hyperpolarized protons of compounds 1 and 2. STD-NMR signal reduction is calculated by taking the ratio of the difference spectra and the off-resonance spectra, as previously reported.^11^ Photo-CIDNP signal reduction is calculated following eq 4. Figure 4C,D shows a clear correlation between STD-NMR and photo-CIDNP signal reduction, as expected from the similar cross-polarization mechanisms driving the signal difference in both experiments. The H_4_ protons of compounds 1 and 2 exhibit the strongest signal decrease in STD and photo-CIDNP NMR experiments. Inspecting the 3D structures of PIN1-compound 1 (PDB code: 3KCE, Figure S5A) and 2 (PDB code: 2XP6, an analog of 2, Figure S5B) complexes reveals that these protons are inserted into the hydrophobic core of the PIN1 binding pocket.

The hydrophobic core is rich in protons from the methyl groups (M130, L122) and aromatic ring (F134), all prone to cross-polarization. The next largest quenching is observed for the H_1_ of compound 1 and the methyl protons of the imidazole ring of compound 2. Both point toward the cationic region of the binding pocket, which is crucial for ligand binding through building a salt bridge between the carboxy moiety of compounds 1 and 2 and the positively charged region of PIN1 (R68, R69, K63). The H_1_ and methyl protons of compounds 1 and 2, respectively, face L61, whose two methyls are ideal for cross-polarization.^43^ Not only is it possible to establish that the signal quenchings from STD and photo-CIDNP-screening experiments are both driven cross-polarization and that they similarly provide the ligand epitopes but it is also possible to correlate these binding epitopes to the structure–activity relationships of protein–fragment complexes.

## Discussion

The hPTP1E is pivotal in essential biological processes, including protein–protein interactions,^46^ signaling pathways,^47^ and apoptosis regulation.^48^ It has crucial functions in protein–protein recognition and protein complex assemblies.^49−51^ The second out of five PDZ domains (PDZ2 domain) in hPTP1E mediates the recognition and interaction with Ras-associated guanine nucleotide exchange factor 2 (RA-GEF2), showing a ligand selection binding mechanism^34^ and having downstream effects on various cellular processes, including proliferation, differentiation, and signaling in response to extracellular stimuli.^33^ Therefore, understanding the residues involved in recognizing RA-GEF2 by the PDZ2 domain of hPTP1E is crucial to understanding its function, regulation, and involvement in signaling pathways.^47^Table 1 summarizes all the *K*_D_ values of the N-terminal tryptophan-labeled peptides derived with CIDNP-*K*_D_ in Figure 2 and competition CIDNP-*K*_D_ in Figure 3 and shows similar *K*_D_ values with [^15^N, ^1^H]-HSQC over the entire range of affinities.

As expected, the affinity of the peptides increases with the length, allowing a comparison between CIDNP-*K*_D_ and [^15^N, ^1^H]-HSQC over a range of almost two orders in affinity (Table 1). Interestingly, the affinity of WVSAV is an order of magnitude higher than WSAV, whereas the addition of the residues Q and E to the peptide only increases the affinity by approximately 2-fold. This difference can be explained by looking at the crystal structure of PDZ2 bound to EQVSAV (PDB code 3LNY),^38^ in which the valine residue provides an additional H-bond with T23. In contrast, the glutamine and glutamic acid residues do not form additional H-bonds. As explained in Figure 4 with the example of PIN1, the individual proton binding curves also provide information about the ligand binding epitope. For WSAV, WVSAV, and WEQVSAV, the H_β_ group has the highest maximal signal reduction (Figure S2) and, thus, amplification factor, indicating that it is closer to the binding pocket. The H_4_ proton of WQVSAV has a higher amplification factor than its H_β_ protons, indicating that the indole ring is engaged in the binding. Indeed, those findings are supported by the results of the competition CIDNP- *K*_D_ assay where the nontryptophan-labeled QVSAV peptide was titrated against WQVSAV. Table 1 shows that WQSAV is a stronger binder to the PDZ2 domain than QVSAV, with an affinity increase of nearly 30-fold, indicating that the tryptophan residue adds beneficial interactions to stabilize the complex. It is important to note that to successfully track AF_*ss*_ reduction during competition CIDNP-*K*_D_ assay, it requires the absence of signal overlap and considerable signal reduction of the CIDNP reporter. Finally, the signal in the competition assay is a ratio of two AF_ss_ measurements, which also propagates the errors of both measurements.

The presented CIDNP-*K*_D_ approach applies to peptide screenings^52^ and macrocycles,^11,53^ including hit validation and affinity constant determination. This approach is agile since tryptophan or other photo-CIDNP-active amino acids, such as tyrosine, histidine, methionine,^29^ or heteroaromatic artificial amino acids, can be introduced into any peptide, making it suitable for diverse photo-CIDNP applications and expanding the accessible chemical space to a large variety of peptide analogs.

On the other hand, the recent introduction of ultrafast photo-CIDNP fragment screening allows the detection of hits within seconds of measurement at low μM ligand and protein concentrations^24^ and even with benchtop NMR spectrometers.^25^ The design of libraries containing a diverse set of several hundreds of molecules^24^ and ongoing efforts to expand this chemical space to several thousands of molecules represent an opportunity for NMR-based fragment screening to increase its throughput considerably. The extension of CIDNP small-molecule screening with the CIDNP-*K*_D_ method allows embedding it directly into a FBDD pipeline. The affinities of the well-characterized PIN1-binding compounds 1 and 2 were obtained using CIDNP-*K*_D_ in agreement with the values reported in the literature.^24,42,54^ Considering the typical hit rates obtained in fragment screening, the automatized CIDNP-*K*_D_ method can determine the affinities of all the hits within one or 2 days, allowing triaging the dozens of fragment hits for follow-up growth or linking.

In addition, comparing similar compounds with different affinities is essential to build a structure–activity relationship rationale, in complement or not, of 3D protein–ligand complex structures.^55^ The reported structures of PIN1 in complexes with compounds 1 and 2 (Figure S4A,B)^43^ confirm that the STD and photo-CIDNP signal quenching is specific to each proton regarding its environment in the binding pocket. More specifically, the strongest signal quenching is observed for the proton H_4_ of both compounds, which inserts into the proton-rich hydrophobic core of PIN1’s binding site (L122, M130, F134). Quenching of H_2_ of 1 and H_6_ of 2 is similar as they occupy the same space near S154. However, in contrast to STD-NMR, photo-CIDNP brings the system up to 100 times more out of the Boltzmann equilibrium than one reaches with the saturation of the protein. This boosts the experimental sensitivity and increases the dynamic range of the detection indicated in Figures 4C,D and S4C–F, where the photo-CIDNP signal quenching is about 10–20 times larger than the STD signal quenching. However, while STD-NMR can yield information about each proton in the molecule, CIDNP only provides information on the protons that are polarizable by photo-CIDNP. This particular feature proves helpful in differentiating between true and false positives due to a pipetting error.^13^

In general, the protein concentrations used in the direct and indirect CIDNP-*K*_D_ measurements for the peptides (20, 10, or 5 μM) and the fragments (20 μM) are lower than the concentrations used in the [^15^N, ^1^H]-HSQC titration experiments (30 μM PDZ2 domain, 80 μM PIN1). In addition, the experimental time per titration point is drastically reduced by using photo-CIDNP hyperpolarization. It took 80 and 30 min per titration point to record [^15^N, ^1^H]-HSQC spectra at a concentration of 30 μM PDZ2 or 80 μM PIN1, respectively. The measurement of one titration point with photo-CIDNP takes only around 30 s. Extrapolating with the sample exchange time in the order of 2–3 min, the throughput of the CIDNP-*K*_D_ approach is 1 order of magnitude faster than other NMR methods (Table S1). In addition to the time savings, the photo-CIDNP experiment does not require isotope labeling, making it much more cost-effective and accessible to targets that require mammalian expression systems. In the future, the gain in sensitivity will allow the use of smaller NMR tubes, such as 1–1.7 mm tubes, which will reduce protein consumption by up to 1 order of magnitude. The counterpart will be the accumulation of several transient experiments to compensate for the smaller volume, but the throughput will remain high, especially thanks to the fact that photo-CIDNP does not require interscan delays.^56^ Furthermore, as the CIDNP-*K*_D_ method is driven by selective longitudinal relaxation and is free from chemical exchange, it is free from nonkinetic components, making the analysis less complex and more reliable in comparison to other ligand-observed NMR affinity determination methods (Table S1).

A drawback of the method is the limitation that it can only be applied to weak binding complexes with affinities higher than roughly 10 μM, where the ligand is in the fast-exchange regime. While in the beginning of a FBDD process, the ligands might be in this regime, lead molecules are typical in the affinity range of nanomolar or even picomolar and other methods like SPR or ITC might be better suited. However, while it has been shown that the affinity of the binder in the nanomolar regime is accessible with ^19^F competition experiments, we assume that this methodology works in the context of competition CIDNP-*K*_D_, which will need further investigation.^57^ Furthermore, there might not always be suitable binders that undergo photo-CIDNP hyperpolarization with sufficient signal-to-noise enhancement, limiting the applicability of CIDNP-*K*_D_.

## Conclusions

In conclusion, we present three other critical steps besides the photo-CIDNP screening: First, the affinity (*K*_D_) determination is within minutes of measurement time, enabling the manual characterization of up to 60 weakly binding hits per day. The complete automation of the photo-CIDNP NMR platform would push the affinity determination throughput up to 150 *K*_D_’s per day. Such throughput performances are, to the best of our knowledge, more than 1 order of magnitude higher than the state of the art. The method does not need isotope labeling and works at low-μM protein concentrations.

Second, previous limitations regarding the chemical space of photo-CIDNP-polarizable molecules are addressed by developing competition experiments that are also quantitative. This demonstrates the possibility of screening nonphoto-CIDNP libraries using a photo-CIDNP handle.

Finally, we show how the selective longitudinal relaxation mechanism opens the door to ligand-binding epitope determination directly from the photo-CIDNP screening results. For example, ambiguous restraints derived from the epitopes could be used in structural methods such as docking,^58^ MD simulations,^59^ CORCEMA,^60^ or other structural approaches for the follow-up procedure of lead compound development.^61^

## Materials and Methods

### PDZ2 and PIN1 Expression and Purification

Expression and purification were carried out as in previously reported procedures. In short, expression in *Escherichia coli* cells (BL21 (DE3)) and purification via Ni-NTA chromatography as the constructs used for the PDZ2 domain of human tyrosine phosphatase 1E and human PIN1 both contained N-terminal polyhistidine tags. Both protein constructs were measured in an NMR buffer consisting of 50 mM NaCl, 20 mM KPO_4_, and 10% D_2_O at pH 6.8. For the control [^1^H, ^15^N]-HSQC experiments, the PDZ2 domain was expressed in ^15^N-enriched minimal medium.^34,43^

### NMR Experiments

All photo-CIDNP and STD-NMR experiments were recorded at 298 K on a Bruker Avance III HD 600 MHz spectrometer equipped with a cryoprobe. The [^15^N, ^1^H] – HSQC titration experiments of the PDZ2 domain were performed also at 298 K on another Bruker Avance IIII HD 600 MHz spectrometer equipped with a cryoprobe and SampleJet.

The laser for the photo-CIDNP experiments was a Thorlabs L450P1600MM with light emitting at 450 nm. An optic fiber (Thorlabs, FG95UEC, 0.95 mm diameter) was inserted into the 3 mm NMR tube ending above the NMR coil region.

To remove oxygen in the NMR tube and prevent photosensitizer quenching, an enzyme cocktail of glucose oxidase (GO, 120 kDa), catalase (CAT, 240 kDa), and d-glucose was used at a concentration of 200 nM, 200 nM, and 5 mM, respectively. 25 μM fluorescein was used in all photo-CIDNP experiments as a photosensitizer.^35^

The photo-CIDNP titration experiments were carried out for the peptide WSAV with 100, 200, 400, 600, 800, and 1600 μM peptide concentration with and without 20 μM PDZ2. The light irradiation durations were 0, 1000, 1500, 4, 6, 8, 10, 14, 18, 22, 26, 50, 100, and 200 ms in this order.

The concentration range of the peptide WVSAV was 10, 25, 50, 100, 150, 200, and 400 μM, and all measurements were carried out with and without 10 μM PDZ2. Light irradiation was for 0, 5, 10, 20, 30, 40, 80, 150, 300, 500, 1000, and 2000 ms.

The concentration range of the peptide WQVSAV was 5, 10, 25, 50, 100, 150, and 400 μM with and without 10 μM PDZ2. The light irradiation was on for 0, 5, 10, 20, 30, 40, 80, 150, 300, 500, 1000, and 2000 ms.

The peptide WEQVSAV was titrated at 5, 10, 25, 50, 100, and 200 μM with and without 10 μM PDZ2. Light irradiation was for 0, 5, 10, 20, 30, 40, 80, 150, 300, 500, 1000, and 2000 ms.

For the photo-CIDNP competition experiment of the peptide QVSAV, a peptide concentration of 0, 20, 40, 80, 150, 300, 500, 1000, and 2000 μM was chosen in the presence of 20 μM peptide WVSAV and 5 μM PDZ. The light irradiation duration was 0, 2, 4, 6, 8, 10, 12, 14, 16, 18, 20, 22, 24, 26, 28, 30, 32, 34, 36, 38, 40, 46, 52, 58, 64, 70, 80, 90, 100, 120, 200, 250, 300, 350, 400, 500, 600, 1000, and 1500 ms.

The competition experiment of the peptide ENEQVSAV was carried out with 10 μM PDZ2, 150 μM peptide WQVSAV, and 0, 10, 20, 40, 60, 80, 100, 150, and 200 μM ENEQVSAV. Light irradiation was for 0, 200, 400, 600, 800, 1000, 1500, and 2000 ms.

The [^15^N, ^1^H]-HSQC experiments for determining the reference affinities were measured for all peptides with 200 (*t*_1,max_ (^15^N) = 48.4 ms) X 2048 (*t*_2,max_ (^1^H) = 121.7 ms) data points with 24 scans per increase and 0.8 s interscan delay. The PDZ2 concentration was 30 μM. The peptides were titrated at the following concentrations: peptides WSAV: 50, 100, 250, 500, 750, 1000, 1500, and 2000 μM, peptide WVSAV: 25, 50, 75, 100, 250, 500, 1000, and 1500 μM, peptide WQVSAV: 10, 25, 50, 75, 100, 250, 500, and 1000 μM, peptide WQVSAV: 5, 10, 25, 50, 75, 200, 250, and 500 μM, peptide WEQVSAV: 5, 10, 25, 50, 75, 100, 250, 500 μM, peptide QVSAV: 25, 50, 75, 100, 250, 500, 1000, and 1500 μM, and peptide ENEQVSAV: 50, 10, 25, 50, 75, 100, 150, and 200 μM. Processing was done with a shifted cosine window function of both dimensions.

PIN1 CIDNP titration experiments were carried out with and without 20 μM PIN1. Compounds 1 and 2 were both titrated with 25, 50, 100, 250, 500, 1000, 1500, and 2000 μM and light irradiation was on for 0, 200, 400, 600, 800, 1000, 1500, and 2000 ms.

STD experiments for compounds 1 and 2 were carried out at a concentration of 500 μM ligand and 20 μM PIN1. Protein saturation was done for 2 s; 32780 (*t*_max_ (^1^H) = 1.7 s) points were measured with 800 scans. The on-resonance pulse was set to −0.5 ppm with the off-resonance pulse set to 60 ppm.

### Data Analysis

Peak picking was performed with the maximal peak picking tool of Mnova software. Plotting, fitting, and data analysis was done in RStudio with the workflow described above. The [^15^N, ^1^H]-HSQC reference affinities were analyzed with CCPNMR3.1.^62^ The backbone assignment was taken from BMRB 34688.

## Data Availability

All data are available upon request.

## Supporting Information Available

Additional experimental results showing the CIDNP-*K*_D_ time dependency, CIDNP signal reduction of peptides upon binding PDZ2, ^15^N-HSQC titration data of PDZ2 and the binding peptides, additional CIDNP-*K*_D_ competition binding curves and the binding pocket of PIN1 in complex with compound 1 and 2 and their corresponding STD-NMR results, and comparison of different NMR methods to obtain the protein–ligand affinity (PDF)
