# Supplementary material for: Rapid Protein–Ligand Affinity Determination by Photoinduced Hyperpolarized NMR
Source: J Am Chem Soc. 2024 Jun 19;146(26):17974–85. doi: 10.1021/jacs.4c04000 (PMC11228983; doi:10.1021/jacs.4c04000)
Supplement: Supplementary file 1 — ja4c04000_si_001.pdf [file ja4c04000_si_001.pdf]

Supplementary Materials for

Rapid Protein-Ligand Affinity Determination by Photo-Induced Hyperpolarized NMR

Matthias Bütikofer[1], Gabriela R. Stadler[1], Harindranath Kadavath[1], Riccardo Cadalbert[1], Felix Torres*[1,2] and Roland Riek*[1]

[1] Institute for Molecular Physical Science
Vladimir Prelog Weg 2, 8093, Zürich, Switzerland
E-mail: roland.riek@phys.chem.ethz.ch

[2] NexMR AG
Wiesenstrasse 10A, 8952, Schlieren, Switzerland
ftorres@nexmr.com

*Correspondence to:
ftorres@nexmr.com; roland.riek@phys.chem.ethz.ch

Photo-CIDNP, Affinity determination, drug discovery, FBDD

This PDF file includes:
Fig. S1 to S5
Table S1

Fig. S1.

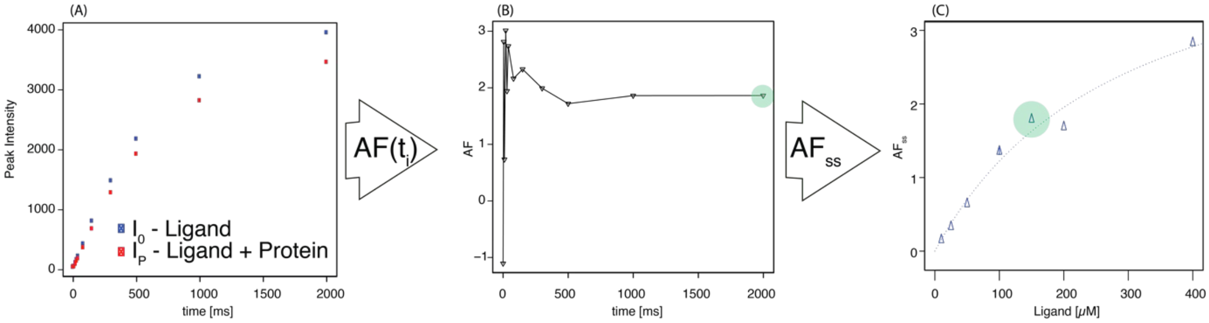

Fig. S1: Data analysis workflow of CIDNP-K_D. A) CIDNP intensity build-up over different irradiation times of the ligand in the absence and presence of the protein. (B) Calculation of the amplification factor $AF(t_i)$ over time using Equation 4. Steady state is reached approximately after 500 ms. (C) The amplification factor at steady state AF_{ss} (green circle) is measured at a series of ligand concentrations and then fitted with Equation 6 to determine the dissociation constant K_D.

Fig. F2.

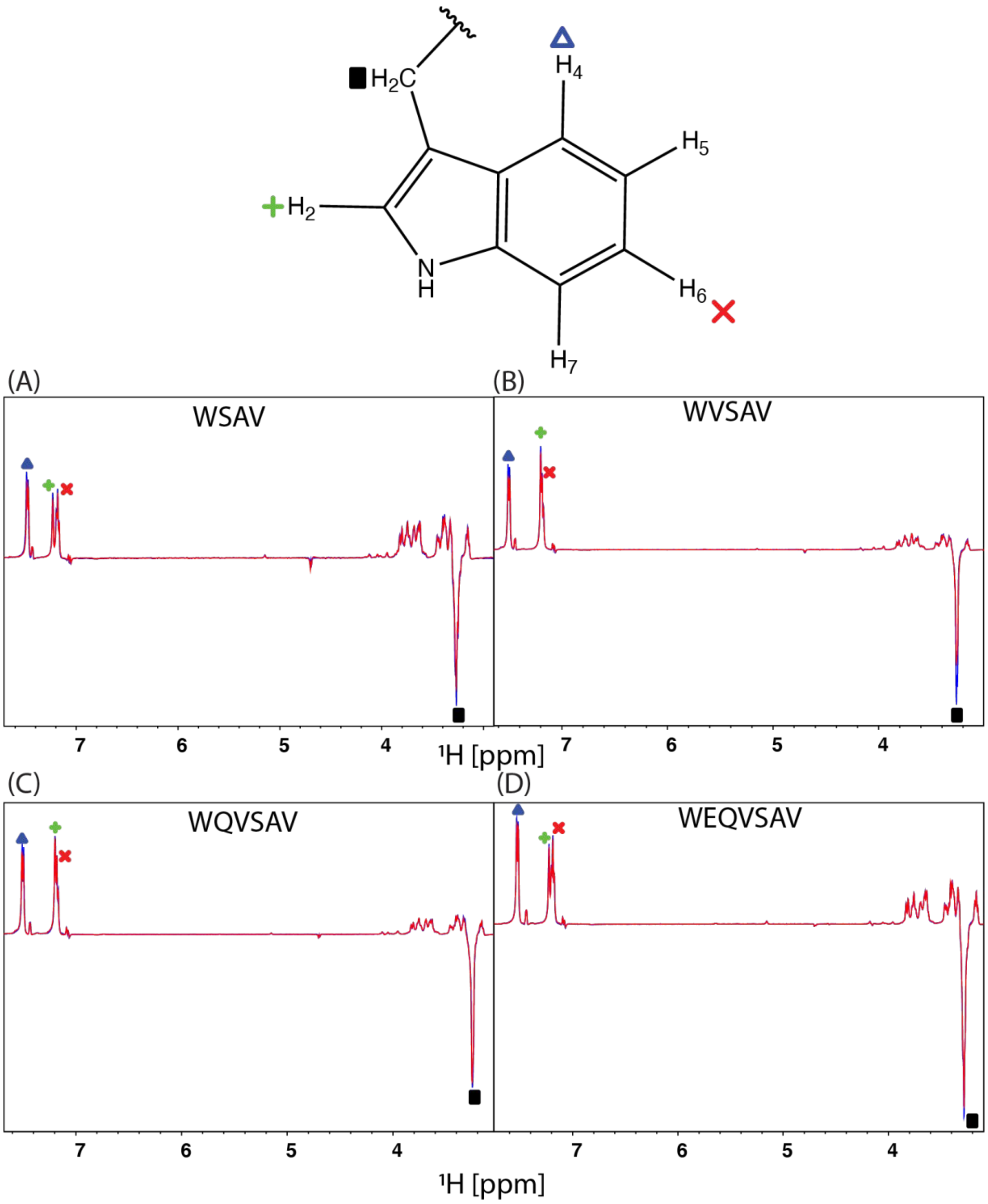

Fig. S2: Photo-CIDNP quenching of peptides upon binding to hPDZ2 domain. 1D photo – CIDNP spectra at 1000 ms laser irradiation of peptides containing a N-terminal tryptophan residue in absence (blue) and presence of hPDZ2 domain (red) is shown for (A) 400 µM WSAV (20 µM hPDZ2), (B) 400 µM WVSAV (10 µM hPDZ2), (C) 400 µM WQVSAV (10 µM hPDZ2), and (D) 200 µM WEQVSAV (10 µM hPDZ2).

Fig. S3.

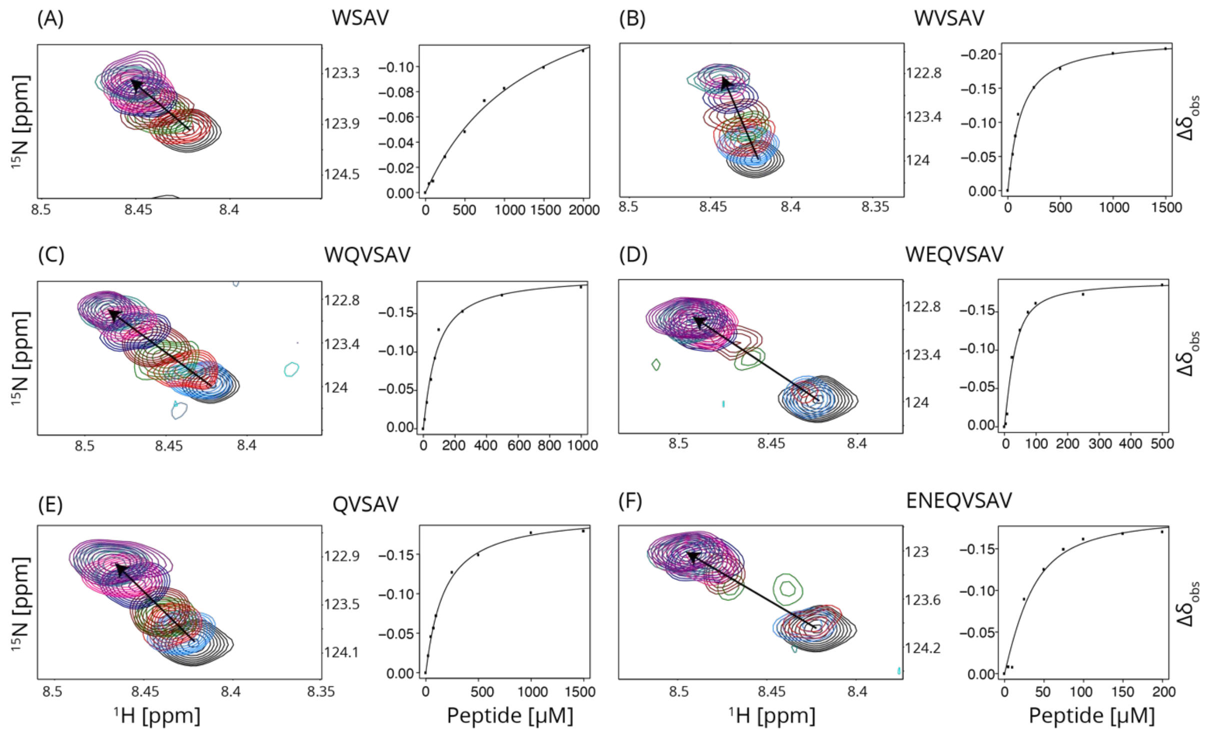

Fig. S3: 2D [^{15}N, ^{1}H]-HSQC titration of peptides to hPDZ2 domain. The 2D [^{15}N, ^{1}H]-HSQC spectra of the titration series in presence of 30 μM hPDZ2 domain and the corresponding observed chemical shift difference of A74 plotted versus the peptide concentration are shown for (A) WSAV 50, 100, 250, 500, 750, 1000, 1500, and 2000 μM, (B) WVSAV 25, 50, 75, 100, 250, 500, 1000, and 1500 μM, (C) WQVSAV 5, 10, 25, 50, 75, 200, 250, and 500 μM, (D) WEQVSAV 5, 10, 25, 50, 75, 100, 250, 500 μM, (E) QVSAV 25, 50, 75, 100, 250, 500, 1000, and 1500 μM, and (F) ENEQVSAV 50, 10, 25, 50, 75, 100, 150, and 200 μM.

Fig. S4.

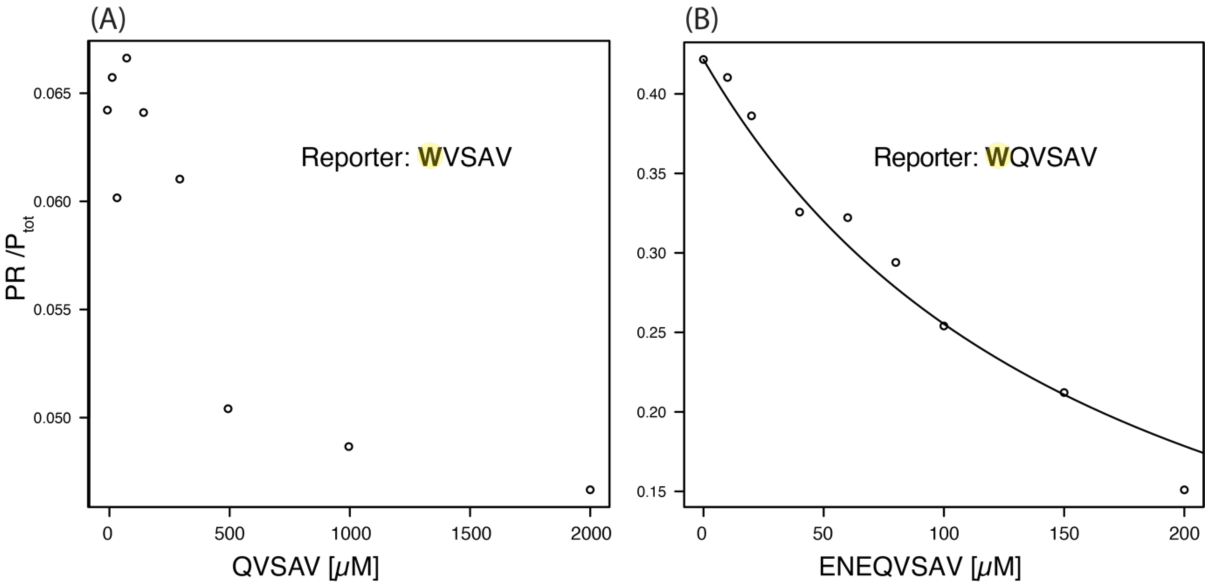

Fig. S4: CIDNP-K_D competition assay to measure binding affinity of non-photo-CIDNP-active peptides binding to a PDZ2 domain. (A) WVSAV (20 μM) bound to human tyrosine phosphatase PDZ2 (5 μM) is replaced by titrating 0, 20, 40, 80, 150, 300, 500, 1000, and 2000 μM QVSAV, respectively, and (B) WQVSAV (150 μM) bound to PDZ2 (10 μM) is replaced by titrating 0, 2.5, 5, 10, 15, 20, 30, 50, 100, 150, and 200 μM ENEQVSAV, respectively. The hyperpolarized signal of the H_4 group from tryptophan is used to calculate the bound population of the reporter ligand using Equation 7 and the affinities of 330 μM and 230 μM of WVSAV and WQVSAV respectively, which were derived by CIDNP-K_D. The bound reporter population is plotted against the competitor concentration and fitted with Equation 8. No fit was derived for (A), as there was too much overlap of the QVSAV signal with the WVSAV signal hindering an exact readout. Each data point is a single scan experiment with a laser irradiation duration of (A) 1000 ms and (B) 2000 ms.

Fig. S5.

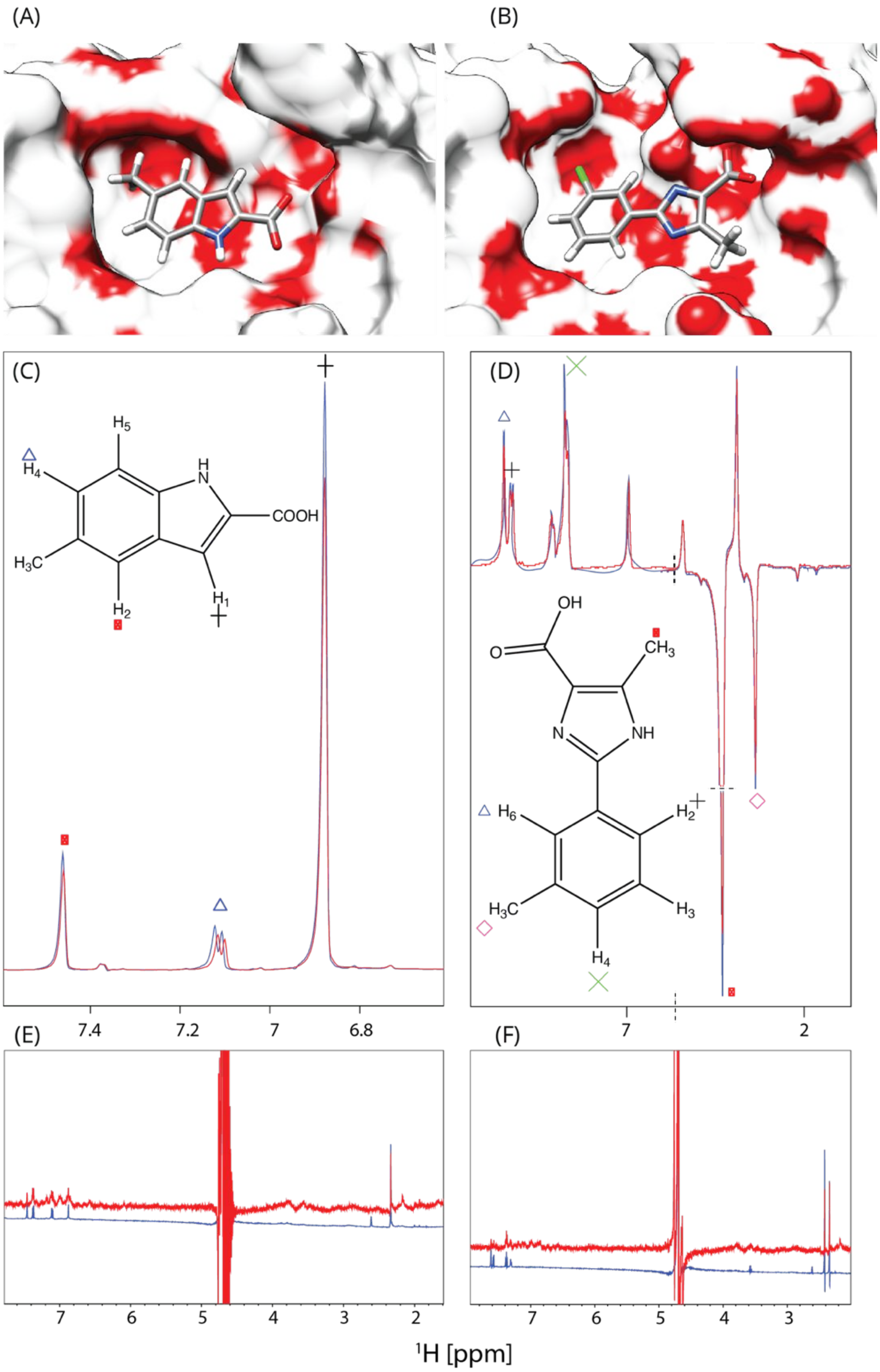

Fig. S5: Complex structure, photo-CIDNP quenching pattern and STD-NMR spectrum of fragments binding to PIN1. PDB structures (A) 3KCE and (B) 2XP6. Protons in 6 Å proximity to the compounds are colored in red, to indicate the spin density around the compounds. The 1D photo-CIDNP spectra of compound 1 (C)and 2 (D) are shown in absence (blue) and presence (red) of 20 µM PIN1 at 500 µM fragment concentration and an irradiation time of 500 ms. The STD-NMR difference spectrum (red) and the STD-NMR off-resonance spectrum (blue) are shown for fragment 1 (E) and 2 (F) at a concentration of 500 µM and PIN1 concentration of 20 µM.

Table. S1: Comparison between different NMR methods to obtain the dissociation constant, assuming 7 titration points per affinity.

Methods	[^{15}N,^{1}H]-HSQC	T_2 relaxation	STD-NMR	CIDNP-K_D
Isotope labeling	Yes	No	No	No
Time per affinity (with autosampler)	2 h	1.5 h	4 h	0.25 h
Typical Protein concentration [µM] / tube	~50	5-10	10-30	5-10
Ligand orientation	No	No	Yes	Yes
Protein binding site	Yes	No	No	No
